# Post-obstructive Diuresis: A Cautionary Tale

**DOI:** 10.7759/cureus.6328

**Published:** 2019-12-08

**Authors:** Amardeep Singh, Bhagwan Dass, Abutaleb Ejaz, Atul Bali

**Affiliations:** 1 Nephrology, University of Florida Health, Gainesville, USA; 2 Nephrology, University of Virginia, Farmville, USA

**Keywords:** nephrogenic, diabetes insipidus, post-obstructive, specific gravity, desmopressin, tadalafil, pde-5, aquaporin, gfr, osmotic diuresis

## Abstract

Post-obstructive diuresis (POD) following decompression of urinary tract obstruction is usually a self-limiting phenomenon. However, prolonged bilateral ureteral obstruction (BUO) can cause severe structural and functional tubular damage. We present a case of POD resulting from partial nephrogenic diabetes insipidus and discuss the diagnosis, treatment, and prognosis.

## Introduction

Post-obstructive diuresis (POD), defined as urine output of 200 mL/hr for two consecutive hours or >3L/24hours, is a polyuric response initiated by the kidneys after the relief of a ureteral obstruction to eliminate accumulated solute and volume. POD occurs in 0.5% to 52% of patients with relief of bilateral ureteral obstruction (BUO) and is usually self-limited [1]. However, prolonged BUO can cause severe structural and functional tubular damage leading to an attenuated response to endogenous antidiuretic hormone.

## Case presentation

A 60-year-old previously healthy male was admitted to the hospital due to a possible syncopal episode. He had a history of intermittent nocturnal enuresis for the past two years. He was occasionally taking tadalafil, a phosphodiesterase type 5 (PDE-5) inhibitor, and had noticed that his urinary symptoms abated during that period. His baseline renal function was suggestive of chronic kidney disease (CKD) stage 3 in prior assessments wherein a serum creatinine of 1.3 mg/dL with estimated glomerular filtration rate (eGFR) 58 was noted. He was diagnosed with bladder outlet obstruction attributed to benign prostatic hyperplasia (BPH) by a urologist two days prior to hospitalization. Office point-of-care-testing serum creatinine was 4.1 mg/dL (eGFR 15 mL/min/m2) and an indwelling Foley catheter was inserted to relieve urinary tract obstruction, empirically. He was discharged home from the outpatient setting with instructions to drink plenty of fluids.

**Figure 1:**
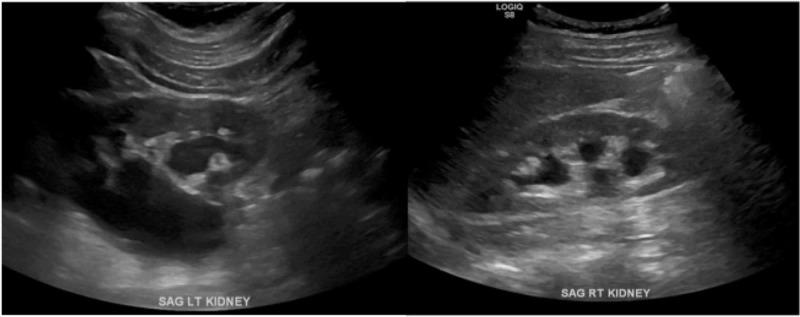
Renal ultrasound showing bilateral hydronephrosis SAG LT KIDNEY: shows a sagittal view of the left kidney; SAG RT KIDNEY: shows a sagittal view of the right kidney.

At home, the patient experienced significant urine output of 15 L over 10-12 hours, associated with intermittent leg cramps which began to worsen in frequency and intensity. The next night, while going to the bathroom, he experienced generalized weakness, diaphoresis, collapsed to the floor and was unresponsive for 20 seconds. He was promptly brought to the emergency department approximately 36 hours after the Foley catheter was placed. On initial examination, vital signs were as follows: temperature 36.6 degrees Centigrade, blood pressure 124/76 mmHg (sitting) and 100/68 mmHg (standing), pulse 78 beats per minute (sitting) and 90 beats per minute (standing), respiratory rate 18/min. The patient was alert and oriented; cardiovascular, respiratory, gastrointestinal, neurological examination were unremarkable. An indwelling Foley catheter bag was present. Laboratory findings included: serum sodium (SNa) 136 mEq/L, potassium 4.2 mEq/L, bicarbonate 21 mEq/L, serum creatinine (SCr) 3.3 mg/dL, blood urea nitrogen (BUN) 49 mg/dL, eGFR 19 mL/min/1.73m2. Urinalysis was unremarkable, other than urine specific gravity (SG) of <1.005. Renal ultrasound demonstrated moderate bilateral hydronephrosis as depicted in Figure 1. The patient was diagnosed with acute kidney injury secondary to obstructive uropathy. Resuscitation with normal saline was initiated.

**Figure 2:**
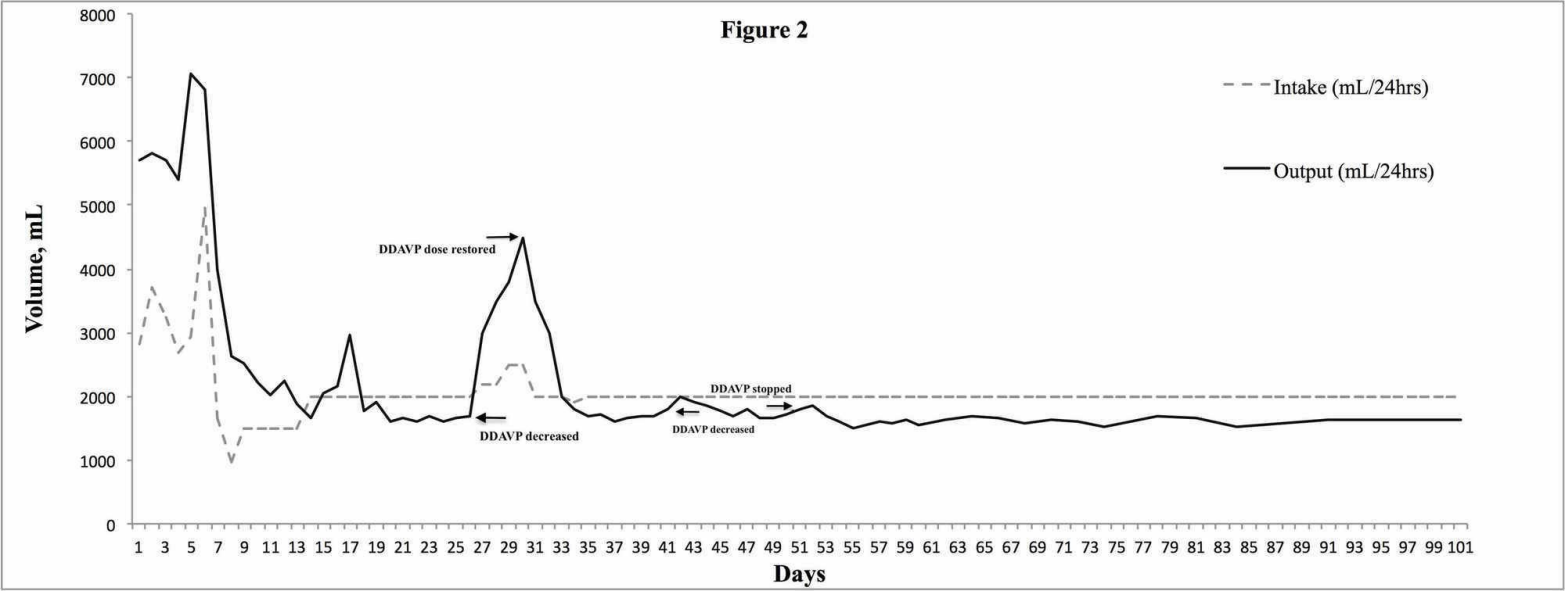
Graph demonstrating the clinical course of the patient DDAVP: desmopressin.

Despite gradual improvement in his clinical status and renal function, he continued to have polyuria (5400 mL/day). Labs now revealed serum osmolality 295 mOsm/kg, urine osmolality 351 mOsm/kg, urine SG of 1.010, pH 7.0. Urine was negative for glucose and protein. Partial nephrogenic diabetes insipidus (NDI) was suspected. The patient was started on nasal desmopressin spray 10 mcg twice daily and was subsequently transitioned to oral desmopressin 0.05 mg tablet twice daily. The clinical course of the patient is shown in Figure 2.

The patient responded to desmopressin with a decrease in urine output and was discharged home. An initial attempt to decrease desmopressin on day 30 to daily dose was unsuccessful as urine output increased dramatically following the change in dose frequency, necessitating restoration of twice-daily administration. A second attempt to decrease desmopressin to a daily dose was successful on day 41 and eventually discontinued on day 50. The patient continued to do well and SCr and eGFR improved to 1.2 mg/dL and 74 mL/min/m2. With close monitoring of fluid intake, SNa remained in the normal range throughout his clinical course. He maintained follow up with his urologist, who performed routine exchanges of his indwelling urinary catheter, while assessing his candidacy for surgical treatment of his BPH. Notably, urodynamic studies were notable for decreased detrusor contractility possibly related to the chronicity of his obstruction, necessitating the maintenance of a chronic indwelling urinary catheter prior to successful transurethral photovaporization of the prostate on day 98. He underwent a voiding trial on post-operative day 3, successfully emptied his bladder, and has been doing clinically well since.

## Discussion

Acute ureteral obstruction is associated with a transient increase in renal blood flow, progressive vasoconstriction, and tubular damage that progresses with chronicity of obstruction. This results in the activation of the renin-angiotensin system, renal prostaglandin synthesis with enhanced production of the vasoconstrictor thromboxane A2. Renal tubular and interstitial cells undergo pronounced apoptosis during the course of chronic BUO. Relief of obstruction is followed by POD, the pathophysiology of which includes a progressive reduction in the medullary concentration gradient secondary to vascular washout and down-regulation of sodium transporters in the thick ascending loop of Henle, reduction in glomerular filtration rate, ischemia and loss of juxtamedullary nephrons associated with vasopressin-resistant impaired renal concentrating ability that can result in partial nephrogenic diabetes insipidus [2]. Tubuloglomerular feedback responses are blunted and sympathetic nerve terminals are damaged by high intrarenal pressure during BUO. Altered regulation of local atrial natriuretic peptide contributes to the functional changes associated with POD after the release of BUO.

Several clinical questions warrant discussion. Would a graduated decompression strategy have resulted in a more favorable outcome for this patient? Quick, complete emptying of the obstructed bladder is safe, simple, and effective and is recommended as the optimal method for decompressing the obstructed urinary bladder, albeit with prudent, supportive care to avoid hypovolemia. In hindsight, our patient would have benefitted from careful preventative measures. 

Osmotic diuresis was a differential diagnostic consideration, defined as urine osmolality >600 mOsm/kg and a normal SNa. Our patient had urine osmolality of 351 mOsm/kg, no glucosuria and responded promptly to desmopressin with increased urine osmolality and decrease urine output, suggesting partial NDI. The patient did not have osmotic diuresis. Brodsky et al. have demonstrated that in osmotic diuresis even the presence of maximal doses of vasopressin fails to increase urine osmolality [3]. Studies have shown that BUO downregulates the expression of vasopressin-sensitive aquaporin 2 (AQP2) water channel in experimental models and represents an important factor in the slow recovery from POD, as in this patient [4].

GFR can decrease by 50% in BUO. Our patient had complete, although delayed, recovery of renal function after relief of obstruction despite the prolonged chronicity and severity of BUO. Preglomerular vasoconstriction may have been the primary mechanism involved in the reduction of GFR. Another important observation was that SNa remained normal throughout the clinical course in the patient despite decreases in eGFR. Studies have shown that in a state of moderate reduction in nephron population there is no evidence that the factors that modulate ion transport are qualitatively different from those that regulate renal function in the intact subject, when the excretory load of solute is varied by changes in intake or endogenous production [5].

It is uncertain whether tadalafil intermittently ameliorated symptoms in our patient. Cyclic guanosine monophosphate (cGMP) mediated signaling pathway also leads to AQP2 membrane insertion. PDE-5 inhibitors elevate intracellular cGMP levels which results in plasma membrane accumulation of AQP2, mimicking vasopressin effect [6].

Were there clinical signs that prognosticated the outcome? Our patient’s urine SG on initial presentation was <1.005, i.e., hypo-osmotic as compared to serum osmolality, and indicated the kidneys’ inability to concentrate urine and consistent with pathologic salt-wasting POD. Random urine sodium levels (not available in this report) >40 mEq/L may also be utilized to suggest renal tubular injury and if prolonged can lead to pathologic POD. The ability to acidify the urine to pH <6.0 preoperatively may be a good predictor of the recovery potential of an obstructed kidney [7]. It is uncertain whether postvoid residual volumes are a predictor of renal outcome.

## Conclusions

In conclusion, urinary tract obstruction can cause a diminished response to endogenous vasopressin resulting in partial nephrogenic diabetes - one of the several mechanisms of polyuria and the hallmark of POD. The prolonged course of polyuria in our patient may be a reflection of the chronicity of his obstruction. Understanding the mechanisms of polyuria in such cases provides therapeutic targets to reduce morbidity and hospitalization.
